# Annular Denuders and Filter Packs Designed to Measure Ambient Levels of Acidic and Basic Air Pollutants

**DOI:** 10.6028/jres.093.046

**Published:** 1988-06-01

**Authors:** Robert K. Stevens

**Affiliations:** U.S. Environmental Protection Agency, Research Triangle Park, NC 27711

Measurements of acidic (e.g., SO_2_, HNO_3_) and basic gases (e.g., NH_3_) that coexist with fine particles (<2.5 *μ*m) are used in models to assist in determining the origin and age of aerosols. Bias associated with each measurement method used to obtain this air quality data can degrade the real correlation between species. In addition, the sensitivity of most instrumental methods to measure SO_2_, NH_3_, HNO_3_ or HNO_2_ is limited to measurements at concentration levels above 2–5 ppb. Measurement of HNO_3_ has also proven to be more difficult due to losses of HNO_3_ in the sampling inlets used in some of the measurement procedures. Difficulties in differentiating atmospheric HNO_3_ from HNO_3_ produced by the dissociation of NH_4_NO_3_ during sampling, further complicates monitoring procedures.

Possanzini et al. [1] described the development of an annular denuder system which removes reactive gases (e.g., HNO_3_, SO_2_) from air samples an order of magnitude more efficiently per unit length and at lower Reynolds number than open tubular denuder designs. Recently we developed an improved version of the annular denuder that incorporates several important features to minimize losses of key species during sampling and reduce the possibility of leaks in the components that join the various parts of the system.

To demonstrate the applicability of this improved design of the annular denuder, a series of field studies were conducted in Research Triangle Park, NC during the fall of 1986 and winter of 1987. An annular denuder system (fig. 1) is composed of four components: an inlet (to remove large particles), coated denuders (to collect the acidic and basic gases), filter pack (to collect fine particles and HNO_3_ that may evaporate from the filter), and a flow controller-pump assembly. In this study a new glass Teflon-coated impactor inlet was designed with a short tube extending below the impactor to prevent large particles and rain droplets from entering the impactor. With the exception of the impactor surface, the entire inlet is coated with Teflon. The impactor surface is a sintered glass disc coated with silicone grease to prevent particle bounce. At 16.7 L/min, a 50% D_AE_=2.64 *μ*m cutpoint was obtained with an impactor nozzle jet diameter of 4.0 mm (Baxter and Lane [2]).

The first two denuder tubes were coated with a 1% solution of glycerine and Na_2_CO_3_. Anions collected on the second denuder are used to correct for any particle deposition that may have occurred in the denuders during sampling. Both denuders were extracted with ion chromatography eluent and analyzed for ${\text{NO}}_{2}^{-}\left({\text{HNO}}_{2}\right),{\text{NO}}_{3}^{-}\left({\text{HNO}}_{3}\right)$, and (SO_2_). The two filters in the filter pack were extracted with 20 mL deionized water and analyzed for ${\text{SO}}_{4}^{=}$ and ${\text{NO}}_{3}^{-}$ content. For some portions of the study, a third annular denuder coated with citric acid was used to collect NH_3_. As a result of these experiments, we have demonstrated that a relatively inexpensive ($150.00) Teflon-coated impactor will quantitatively transmit acidic gases to an annular denuder.

In addition, modifications to the annular denuder itself resulted in a reliable system for field investigations. Paired samples were run to compare the new denuder assemblies and the average percent differences between sampler results with different inlet configurations for SO_2_, HNO3, HNO_2_. The differences were 3.4, 6.4, and 8.3%, respectively. During the second phase of the testing, an NH_3_ denuder was incorporated into the assembly. The average % difference between two identical annular denuder systems for SO_2_, HNO_2_, HNO_3_, NH_3_, ${\text{SO}}_{4}^{=}$ and ${\text{NO}}_{3}^{-}$ were 1.8, 5.5, 15, 16, 4.6 and 3.6%, respectively. Experiments by Appel et al. [3] indicated that HNO_3_ was not retained in the Teflon-coated glass inlets.

In our study, intercomparison of denuder assemblies showed that ratios of HNO_3_ to particle nitrate tended to decrease with decreasing ambient temperature and increasing humidity. This is qualitatively consistent with previous theoretical phase equilibrium calculations of NH_4_NO_3_.

## Figures and Tables

**Figure 1:**
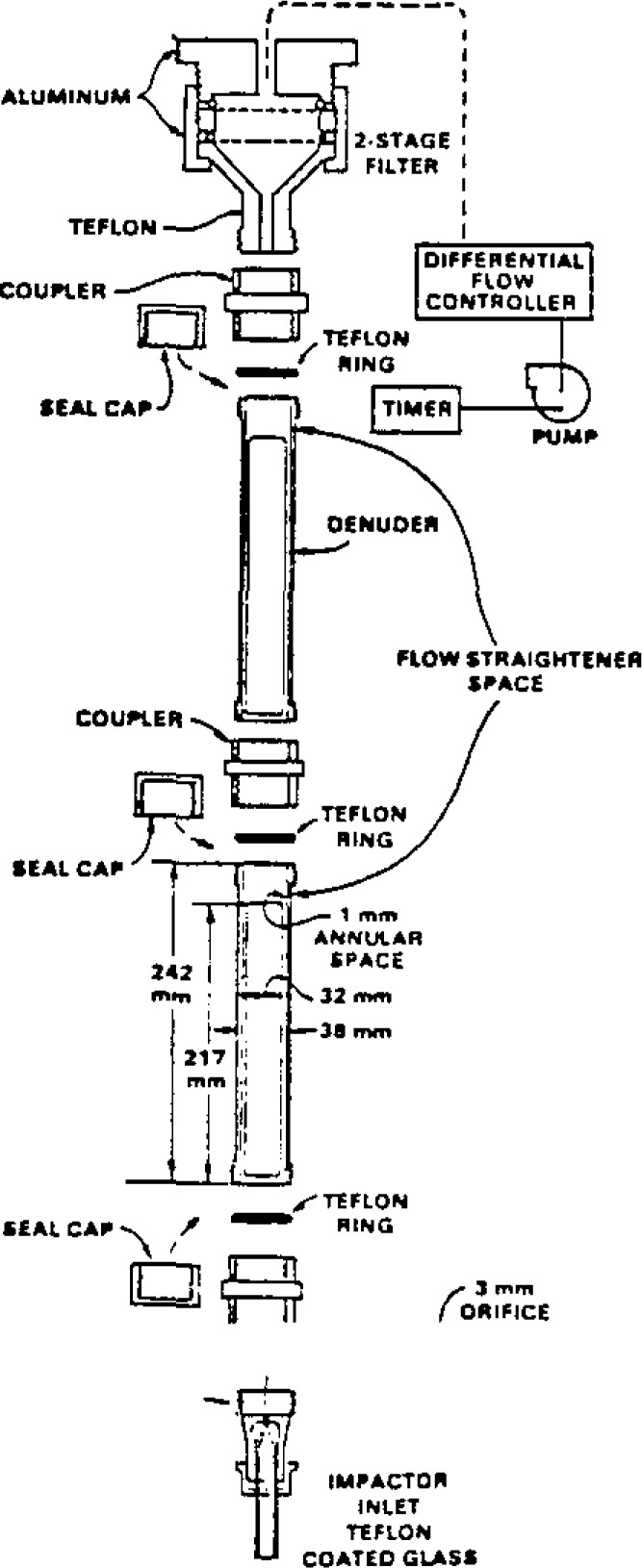
Annular denuder.
